# Monounsaturated Fatty Acid Levels May Not Affect Cardiovascular Events: Results From a Mendelian Randomization Analysis

**DOI:** 10.3389/fnut.2020.00123

**Published:** 2020-09-02

**Authors:** Mohsen Mazidi, Niki Katsiki, Niloofar Shekoohi, Maciej Banach

**Affiliations:** ^1^Department of Twin Research and Genetic Epidemiology, King's College London, London, United Kingdom; ^2^Division of Endocrinology and Metabolism, First Department of Internal Medicine, Diabetes Center, Medical School, AHEPA University Hospital, Thessaloniki, Greece; ^3^Department of Cellular and Molecular Nutrition, School of Nutritional Sciences and Dietetics, Tehran University of Medical Sciences, Tehran, Iran; ^4^Department of Hypertension, Medical University of Lodz, Łódz, Poland; ^5^Polish Mother's Memorial Hospital Research Institute (PMMHRI), Łódz, Poland; ^6^Cardiovascular Research Centre, University of Zielona Gora, Zielona Gora, Poland

**Keywords:** mendelian randomization, serum monounsaturated fatty acids, coronary heart disease, myocardial infarction, cardioembolic stroke, ischemic stroke

## Abstract

**Background/Aim:** Several observational studies evaluated the links between serum monounsaturated fatty acids (MUFAs) and cardiovascular events with controversial results. In the present study, Mendelian randomization (MR) analysis was applied to obtain unconfounded estimates of the causal associations of genetically determined serum MUFAs with coronary heart disease (CHD), myocardial infarction (MI), cardioembolic stroke (CS), and ischemic stroke (IS).

**Methods:** Four MUFAs were studied (i.e., 10-heptadecenoate, myristoleic, oleic, and palmitoleic acid). Data from the largest genome-wide association studies on MUFAs, CHD, MI, and stroke were analyzed. Inverse variance weighted method (IVW), weighted median (WM)-based method, MR-Egger, as well as MR-pleiotropy residual sum and outlier were applied. To rule out the impact of single-nucleotide polymorphism (SNP), the leave-one-out method was also performed.

**Results:** Genetically higher-serum 10-heptadecenoate levels did not affect the risk of CHD (IVW = Beta: −0.304, *p* = 0.185), MI (IVW = Beta: −0.505, *p* = 0.066), CS (IVW = Beta: −0.056, *p* = 0.945), and IS (IVW = Beta: −0.121, *p* = 0.767). Similarly, no significant associations were observed for myristoleic acid (CHD: IVW = Beta: 0.008; MI: IVW = Beta: 0.041; CS: IVW = Beta: 0.881; IS: IVW = Beta: 0.162), oleic acid (CHD: IVW = Beta: −0.2417; MI: IVW = Beta: −0.119; CS: IVW = Beta: 1.059; IS: IVW = Beta: 0.008491), and palmitoleic acid (CHD: IVW = Beta: −0.06957; MI: IVW = Beta: −0.01255; CS: IVW = Beta: 1.042; IS: IVW = Beta: −0.1862). A low likelihood of heterogeneity and pleiotropy was reported, and the observed associations were not driven by single SNPs.

**Conclusions:** In the present MR analysis, serum MUFA levels were not associated with the risk of CHD, MI, CS, and IS. Further research, evaluating more MUFAs, is required to elucidate the links between MUFAs and CVD to contribute to health policy decisions in reducing CVD risk.

## Introduction

Monounsaturated fatty acids (MUFAs) are a subgroup of fatty acids containing a single double bond, with two forms of configuration: *cis* and *trans* (1). Major sources of exogenous MUFAs include vegetable oils, high-fat fruits (such as olives and avocado), red meat, milk products, and nuts (2). MUFAs are also endogenously synthesized in the liver and the adipose tissue by microsomal stearoyl-CoA desaturase-1 (SCD1) from their saturated fatty acid–acetyl-coenzyme A precursors (3).

It is widely known that fatty acid metabolism and composition can be altered during diseases, resulting in beneficial (4, 5) or adverse events (6, 7). In this context, cardiovascular (CV) health may be affected by MUFAs through effects on various markers associated with CHD (8), including serum lipid and lipoprotein profile (9), vascular function markers (10), and postprandial vascular function (11). Indeed some sources of MUFA, such as plant oils, may decrease the risk of CV disease (CVD) in the general population (12). For example, supplemented palmitoleate beneficially influenced the lipid profile [i.e., decreased triglycerides and low-density lipoprotein cholesterol (LDL-C) and increased high-density lipoprotein cholesterol (HDL-C)] as well as reduced the high-sensitivity C-reactive protein (CRP) levels of adults with dyslipidemia (13). Furthermore, circulating palmitoleate levels positively correlated with insulin sensitivity (14).

A significant association between dietary MUFAs and CV risk has been suggested, but the underlying mechanisms are not fully understood yet. A possible explanation may be that endogenously synthesized MUFAs are the major substrate for hepatic triacylglycerol (TAG) synthesis (15), a CVD risk factor (16). Furthermore, serum MUFAs are associated with inflammation and particularly with serum CRP levels (17, 18). Consequently, serum MUFAs can lead to CVD *via* promoting the inflammation process (15).

The activity of SCD1 is enhanced in various cardiometabolic diseases, including diabetes, obesity, and atherosclerosis (19). The increased activity of SCD1 was reported to promote endogenous MUFA synthesis in patients with chronic kidney disease (CKD) (15). In these patients, the serum MUFA levels were positively related to TAG, CRP, and CKD progression and negatively related to HDL-C and LDL-C (15). Furthermore, MUFAs were a strong predictor of CVD incidence in CKD patients (15).

Overall, there is conflicting and limited evidence in relation to the links between serum MUFAs and CVD. The aim of the present study was to evaluate the causal associations of genetically determined serum MUFAs with coronary heart disease (CHD), myocardial infarction (MI), cardioembolic stroke (CS), and ischemic stroke (IS) by applying Mendelian randomization (MR) analysis on data from the largest genome-wide association studies (GWAS) on MUFAs.

## Methods

### Study Design

A two-sample MR study design was used, in which summary statistics from different GWAS were analyzed for the exposures (serum MUFAs) and outcomes (CHD, MI, CS, and IS), to estimate the effects of exposure on outcome (20). Essentially, we applied methods to estimate the unbiased effect of genetic predictors of serum MUFAs to extensively genotyped case–control studies of CHD, MI, CS, and IS.

### Genetic Predictors of Exposures

We retrieved summary data for the associations between single-nucleotide polymorphisms (SNPs) and four circulating MUFAs [i.e., 10-heptadecenoate (17:1), myristoleic acid (14:1; tetradecenoic), oleic acid (18:1; octadecenoic), and palmitoleic acid (16:1; hexadecenoic)] from the GWAS (including 7,824 adult samples of European ancestry) (Supplementary Table 1). Genotyping, quality control, and imputation procedures are described elsewhere (21). If a SNP was unavailable for the outcome GWAS summary statistics, we identified proxy SNPs with a minimum linkage disequilibrium (LD) *r*^2^ = 0.8. To minimize bias in effect estimates induced by the correlation between SNPs, we restricted our genetic instrument to independent SNPs not in LD (*p* = 0.0001). We refer to a set of SNPs that proxy serum MUFAs as “genetic instruments.”

### Genetic Predictors of Outcomes

Genetic associations with CHD were obtained from the largest publicly available extensively genotyped CHD case (*n* ≤ 76,014)–control (*n* ≤ 264,785) study based on a meta-analysis, with the use of double genomic control correction, of the CARDIoGRAMplusC4D 1000 Genomes case(*n* = 60,801)–control (*n* = 123,504) study, the UK Biobank SOFT CAD study (cases *n* = 10,801, controls *n* = 137,371), and two small case (*n* = 4,120)–control (*n* = 3,910) studies from Germany and Greece (22). The CARDIoGRAMplusC4D 1000 Genomes participants are largely of European descent (77%) with detailed phenotyping of CHD, MI, or both based on medical records, clinical diagnosis, and procedures that indicate CHD (22, 23).

Genetic associations with different stroke types were obtained from the largest available extensively genotyped dataset, i.e., the METASTROKE, a collaboration of the International Stroke Genetics Consortium, including GWAS data on 34,217 ischemic cases and 404,630 controls of European ancestry from across 15 countries (24). The majority of the cases involved brain imaging confirmation. Additional phenotype descriptions and details of individual studies, including data collection and genetic data quality control procedures, are reported elsewhere (24).

### Statistics

We combined the effect of instruments using inverse variance weighted (IVW) method. To address the potential effect of pleiotropic variants on the final effect estimate, we performed sensitivity analysis, including weighted median (WM) and MR-Egger. The sensitivity analysis was conducted using the leave-one-out method to identify instruments that might drive the MR results. The WM estimate provides correct estimates as long as SNPs accounting for ≥50% of the weight are valid instruments. Inverse variance is used to weight the variants and bootstrapping is applied to estimate the confidence intervals (CIs) (20). MR-Egger is able to make estimates even under the assumption that all SNPs are invalid instruments, as long as the assumption of instrument strength independent of direct effect (InSIDE) is satisfied (20). However, the InSIDE assumption cannot be easily verified. Average directional pleiotropy across genetic variants was assessed from the *p*-value of the intercept term from MR-Egger (20). Furthermore, causal estimates in MR-Egger are less precise than those obtained by using IVW MR (25). The analysis using MR-Egger has a lower false-positive rate but a higher false-negative rate than IVW, i.e., it has a lower statistical power (26).

Heterogeneity between individual genetic variant estimates was assessed by the use of the *Q*′ heterogeneity statistic (27). The *Q*′ statistic uses modified second-order weights that are a derivation of a Taylor series expansion, taking into account the uncertainty in both the numerator and the denominator of the instrumental variable ratio (27). Linkage disequilibrium (LD) clumping (0.0001) was applied on all SNPs with *p*-value less than GWAS level.

### Sensitivity Analysis

MR-Egger and MR pleiotropy residual sum and outlier (MR-PRESSO) tests were used for sensitivity analysis (27). MR-Egger and MR-PRESSO may provide correct estimates as long as the instrument strength independent of direct effect assumption is satisfied (26). MR-Egger can be imprecise, particularly if the associations for SNPs on exposure are similar or the number of genetic instruments is low (27). A non-null MR-Egger intercept suggests that the IVW estimate is invalid. MR-Egger does not explicitly identify outliers. MR-PRESSO detects and, if necessary, corrects for potentially pleiotropic outliers (27). The MR-PRESSO framework detects effect estimates that are outliers and removes them from the analysis by regressing the variant–outcome associations on variant–exposure associations. A global heterogeneity test is then implemented to compare the observed distance between residual sums of squares of all variants to the regression line with the distance expected under the null hypothesis of no pleiotropy (28).

MR-robust adjusted profile score (RAPS) was also applied. This method can correct for pleiotropy using robust adjusted profile scores. We consider, as results, causal estimates that agreed in direction and magnitude across MR methods, passed nominal significance in IVW MR, and did not show evidence of bias from horizontal pleiotropy using heterogeneity tests. All analyses were performed using the R software (version 3.4.2 R Core Team, 2017).

### Ethics

The present analysis uses published or publicly available summary data. No original data were collected for this study. Ethical approval for each of the studies included in the present analysis can be found in the original publications (including informed consent from each participant). The study conforms to the ethical guidelines of the 1975 Declaration of Helsinki.

## Results

The instrument associations for serum MUFA levels are shown in the Supplementary Table 1. The instruments have F-statistics higher than threshold, making significant bias from the use of weak instruments unlikely (29). The results, expressed as Beta-coefficient for serum MUFAs per one standard deviation (SD) increase in outcomes, are presented in Tables 1–4.

**Table 1:** Results of the Mendelian Randomization (MR) analysis for 10-heptadecenoate (17:1) and cardiovascular events.

**Exposures**	**MR**				**Heterogeneity**			**Pleiotropy**		
	**Method**	**beta**	**SE**	***p***	**Method**	**Q**	**p**	**Intercept**	**SE**	***p***
Coronary heart disease	MR Egger	0.8635	1.306	0.5447	MR-Egger	2.963	0.558	−0.023	0.025	0.415
	WM	−0.2906	0.2939	0.3228						
	IVW	−0.3047	0.23	0.1852	IVW	3.863	0.576			
	RAPS	−0.298	0.245	0.2239						
Myocardial infarction	MR Egger	2.281	1.457	0.1925	MR-Egger	2.003	0.736	−0.054	0.028	0.124
	WM	−0.2342	0.3345	0.4838						
	IVW	−0.505	0.2754	0.06677	IVW	5.774	0.362			
	RAPS	−0.544	0.304	0.07355						
Cardioembolic stroke	MR Egger	−2.272	4.623	0.6488	MR-Egger	3.426	0.485	0.043	0.088	0.652
	WM	−0.4173	1.073	0.6973						
	IVW	−0.05633	0.8168	0.945	IVW	3.725	0.596			
	RAPS	−0.1773	0.8609	0.8368						
Ischemic stroke	MR Egger	1.04	2.317	0.6769	MR-Egger	1.266	0.865	−0.022	0.044	0.638
	WM	−0.09585	0.5161	0.8527						
	IVW	−0.121	0.4098	0.7679	IVW	1.595	0.911			
	RAPS	−0.1232	0.4288	0.7738						

*WM, Weighted median; IVW, Inverse variance weighted; SE, standard error; beta, beta-coefficients; MR, Mendelian randomization; RAPS, Robust Adjusted Profile Score*.

**Table 2:** Results of the Mendelian Randomization (MR) analysis for Myristoleic acid (14:1) and cardiovascular events.

**Exposures**	**MR**				**Heterogeneity**			**Pleiotropy**		
	**Method**	**beta**	**SE**	***p***	**Method**	**Q**	**p**	**Intercept**	**SE**	***p***
Coronary heart disease	MR Egger	0.3032	0.8636	0.759	MR-Egger	1.623	0.446	−0.0074	0.021	0.755
	WM	0.06446	0.319	0.8398						
	IVW	0.008949	0.2636	0.9729	IVW	1.742	0.623			
	RAPS	0.00909	0.2757	0.9737						
Myocardial infarction	MR Egger	−0.2609	0.9332	0.8061	MR-Egger	1.369	0.526	0.0076	0.022	0.766
	WM	0.03509	0.3512	0.9204						
	IVW	0.0411	0.2897	0.8872	IVW	1.475	0.692			
	RAPS	0.04163	0.3019	0.8903						
Cardioembolic stroke	MR Egger	−1.429	3.887	0.7485	MR-Egger	2.145	0.346	0.054	0.089	0.602
	WM	0.3764	1.205	0.7547						
	IVW	0.8811	0.942	0.3496	IVW	2.536	0.475			
	RAPS	0.902	1.001	0.3677						
Ischemic stroke	MR Egger	0.3005	1.82	0.8841	MR-Egger	1.236	0.536	−0.0033	0.042	0.945
	WM	−0.04136	0.5576	0.9409						
	IVW	0.1628	0.4678	0.7278	IVW	1.425	0.742			
	RAPS	0.1649	0.4878	0.7354						

*WM, Weighted median; IVW, Inverse variance weighted; SE, standard error; beta, beta-coefficients; MR, Mendelian randomization; RAPS, Robust Adjusted Profile Score*.

**Table 3:** Results of the Mendelian Randomization (MR) analysis for Oleic acid (18:1) and cardiovascular events.

**Exposures**	**MR**				**Heterogeneity**			**Pleiotropy**		
	**Method**	**beta**	**SE**	***p***	**Method**	**Q**	**p**	**Intercept**	**SE**	***p***
Coronary heart disease	MR Egger	−2.516	2.943	0.4828	MR-Egger	3.695	0.156	0.034	0.043	0.514
	WM	−0.5437	0.5185	0.2944						
	IVW	−0.2417	0.5068	0.6334	IVW	4.856	0.189			
	RAPS	−0.3834	0.4266	0.3687						
Myocardial infarction	MR Egger	−2.551	2.849	0.4651	MR-Egger	2.756	0.246	0.036	0.042	0.477
	WM	−0.3194	0.5727	0.5771						
	IVW	−0.119	0.502	0.8125	IVW	3.856	0.286			
	RAPS	−0.2092	0.4717	0.6574						
Cardioembolic stroke	MR Egger	−10.12	7.623	0.3155	MR-Egger	0.418	0.812	0.16	0.11	0.274
	WM	1.591	1.757	0.365						
	IVW	1.059	1.378	0.4419	IVW	2.693	0.469			
	RAPS	1.088	1.464	0.4572						
Ischemic stroke	MR Egger	−1.607	3.805	0.7139	MR-Egger	0.736	0.695	0.024	0.055	0.708
	WM	0.06184	0.7951	0.938						
	IVW	0.008491	0.6867	0.9901	IVW	0.923	0.823			
	RAPS	0.008555	0.711	0.9904						

*WM, Weighted median; IVW, Inverse variance weighted; SE, standard error; beta, beta-coefficients; MR, Mendelian randomization; RAPS, Robust Adjusted Profile Score*.

**Table 4:** Results of the Mendelian Randomization (MR) analysis for Palmitoleic acid (16:1) and cardiovascular events.

**Exposures**	**MR**				**Heterogeneity**			**Pleiotropy**		
	**Method**	**beta**	**SE**	***p***	**Method**	**Q**	**p**	**Intercept**	**SE**	***p***
Coronary heart disease	MR Egger	0.6596	0.6251	0.402	MR-Egger	0.336	0.845	−0.022	0.017	0.331
	WM	−0.1111	0.3084	0.7186						
	IVW	−0.06957	0.2514	0.782	IVW	1.956	0.586			
	RAPS	−0.07158	0.2659	0.7878						
Myocardial infarction	MR Egger	0.5029	0.7292	0.5617	MR-Egger	0.485	0.786	−0.015	0.02	0.523
	WM	0.07973	0.3459	0.8177						
	IVW	−0.01255	0.2845	0.9648	IVW	1.078	0.782			
	RAPS	−0.01271	0.2963	0.9658						
Cardioembolic stroke	MR Egger	−5.925	4.205	0.2942	MR-Egger	1.163	0.559	0.17	0.098	0.230
	WM	1.211	1.266	0.3385						
	IVW	1.042	1.16	0.3687	IVW	4.075	0.256			
	RAPS	1.191	1.073	0.2667						
Ischemic stroke	MR Egger	−2.481	1.977	0.3362	MR-Egger	1.775	0.415	0.056	0.047	0.354
	WM	−0.3666	0.6421	0.5681						
	IVW	−0.1862	0.5075	0.7137	IVW	3.265	0.326			
	RAPS	−0.1927	0.5236	0.7129						

*WM, Weighted median; IVW, Inverse variance weighted; SE, standard error; beta, beta-coefficients; MR, Mendelian randomization; RAPS, Robust Adjusted Profile Score*.

Genetically higher-serum 10-heptadecenoate levels had no significant effect on the risk of CHD (IVW = Beta: −0.304, *p* = 0.185, Table 1), MI (IVW = Beta: −0.505, *p* = 0.066, Table 1), CS (IVW = Beta: −0.056, *p* = 0.945, Table 1), and IS (IVW = Beta: −0.121, *p* = 0.767, Table 1). Similar results were obtained for myristoleic acid (for CHD: IVW = Beta: 0.008, *p* = 0.972; for MI: IVW = Beta: 0.041, *p* = 0.887; for CS: IVW = Beta: 0.881, *p* = 0.349; for IS: IVW = Beta: 0.162, *p* = 0.727, Table 2), oleic acid (for CHD: IVW = Beta: −0.2417, *p* = 0.6334; for MI: IVW = Beta: −0.119, *p* = 0.8125; for CS: IVW = Beta: 1.059, *p* = 0.4419; for IS: IVW = Beta: 0.008491, *p* = 0.9901, Table 3), and palmitoleic acid (for CHD: IVW = Beta: −0.06957, *p* = 0.782; for MI: IVW = Beta: −0.01255, *p* = 0.9648; for CS: IVW = Beta: 1.042, *p* = 0.3687; for IS: IVW = Beta: −0.1862, *p* = 0.7137, Table 4).

Heterogeneity results and pleiotropy bias are also shown in Tables 1–4. Estimation based on both MR Egger and IVW was higher than 0.05, indicating low heterogeneity (all IVW *p* > 0.256, all MR Egger *p* > 0.156). Furthermore, MR-PRESSO analysis did not indicate any outliers for all estimates. The horizontal pleiotropy test, with very negligible Egger regression intercept, also showed a low likelihood of pleiotropy for all of our estimations (all *p* > 0.124). The results of the MR-RAPS were identical with the IVW estimates, highlighting again a low chance of pleiotropy. The leave-one-out method demonstrated that the associations were not driven by single SNPs.

## Discussion

The present MR analysis showed that genetically higher-serum MUFA (10-heptadecenoate, myristoleic, oleic, and palmitoleic acids) levels did not affect the risk of CHD, MI, CS, and IS. These associations were not driven by single SNPs, and there was a low chance of heterogeneity and pleiotropy.

Heptadecanoic acid was inversely associated with the risk for MI in 385 women in a population-based cohort (OR = 0.74, 95% CI: 0.58–0.95); in a multivariate analysis, however, this link became non-significant (30). Furthermore, among 2,907 US adults followed up for 22 years, no significant associations were observed between heptadecanoic acid and CVD, CHD, or stroke (31). Similarly, heptadecanoic acid level was not related to the risk of total stroke, IS, or hemorrhagic stroke in the Health Professionals Follow-Up Study (51,529 men) and the Nurses' Health Study (121,700 women) (32). However, a previous meta-analysis (13 prospective studies; 7,680 CVD cases) showed a marginal inverse correlation between heptadecanoic acid and CVD events (relative risk = 0.82, 95% CI: 0.68–0.99) (33). Data on the associations of myristoleic acid with CVD are missing.

Plasma oleic acid was a predictor for CVD events (HR = 1.41; *p* = 0.008) among 6,568 participants of the Multi-Ethnic Study of Atherosclerosis (34). Furthermore, the multivariable-adjusted OR for IS related to a one-SD increment in serum oleic acid was 1.20 (95% CI: 1.01–1.43) in a nested prospective case–control study involving participants from the Women's Health Initiative Observational Study (35). In contrast, oleic acid was inversely associated with acute MI (OR = 0.42; *p* = 0.03) in a case–control study (*n* = 297) (36). Furthermore, there is evidence that oleic acid may act protectively against stroke (37). In this context, higher plasma oleic acid was related to 73% lower stroke incidence (95% CI: 10–92%; *p* = 0.03) among 1,245 participants from the Three-City Study followed up for a median of 5.25 years (38).

Palmitelaidic acid was related to a lower risk of CVD after adjusting for age, gender, and race in a cross-sectional study using data from the National Health and Nutrition Examination Survey 1999–2000 and 2009–2010 (39). Similarly, palmitic acid was inversely associated with acute MI (OR = 0.58; *p* = 0.03) in a case–control study (*n* = 297) (36). In contrast, a high content of palmitoleic acid was suggested as a marker of increased CHD risk (40). In this context, in a prospective nested case–control study of 12,840 participants from the Circulatory Risk in Communities Study, the multivariate OR of coronary artery disease for the highest vs. the lowest quartiles of palmitoleic acid was 3.2 (95% CI: 1.7–6.1) (41). Furthermore, positive linear associations were observed between palmitoleic acid and IS [for plasma cholesterol ester fraction: HR = 1.86 (95% CI: 1.20–2.87); *p* = 0.003; for phospholipid fraction: HR = 1.52 (95% CI: 0.99–2.34); *p* = 0.005] in 3,870 participants from the Minneapolis field center of the Atherosclerosis Risk in Communities study (35). Similarly, a previous meta-analysis (13 prospective studies; *n* = 7,680 CVD cases) found no association between trans-palmitoleic acid, CHD, and stroke (33). Trans-palmitoleic acid levels did not correlate with the risk of total stroke, IS, or hemorrhagic stroke in two large cohort studies [i.e., the Health Professionals Follow-Up Study (51,529 men) and the Nurses' Health Study (121,700 women)] (32). Finally, in another meta-analysis including 32 observational studies of dietary fatty acids (512,420 participants) and 27 randomized clinical trials of fatty acid supplementation (105,085 participants), no associations were observed between fatty acids and coronary disease (42).

In conclusion, the present MR analysis showed that serum MUFA levels did not correlate with the risk of CHD, MI, CS, and IS. Further studies, investigating also other MUFAs, are needed to clarify the links between MUFAs, CHD, MI, CS, and IS. This research is clinically important for health policy decisions in reducing CVD risk.

## Data Availability Statement

All datasets presented in this study are included in the article/Supplementary Material.

## Author Contributions

All authors contributed to the design, interpretation, and writing of the paper.

## Conflict of Interest

NK has given talks, attended conferences, and participated in trials sponsored by Astra Zeneca, Bausch Health, Boehringer Ingelheim, Elpen, Menarini, Mylan, NovoNordisk, Sanofi, and Servier. The remaining authors declare that the research was conducted in the absence of any commercial or financial relationships that could be construed as a potential conflict of interest.
