# Supplementary material for: Monounsaturated Fatty Acid Levels May Not Affect Cardiovascular Events: Results From a Mendelian Randomization Analysis
Source: Front Nutr. 2020 Sep 2;7:123. doi: 10.3389/fnut.2020.00123 (PMC7492609; doi:10.3389/fnut.2020.00123)
Supplement: Supplementary file 1 [file Table_1.docx]

| **Supplementary Table 1.** Summary results of the genetic loci of monounsaturated fatty acids (MUFAs) | | | | | | |
| --- | --- | --- | --- | --- | --- | --- |
| **Traits** | **SNP** | **GX** | **GX SE** | **EA** | **OA** | **EAF** |
| 10-heptadecenoate (17:1) | rs220992 | 0.0155 | 0.0033 | a | g | 0.4501 |
|  | rs1822906 | 0.0188 | 0.0041 | a | g | 0.205 |
|  | rs4288115 | -0.0172 | 0.0038 | a | t | 0.3119 |
|  | rs10098688 | 0.0242 | 0.0054 | a | c | 0.3373 |
|  | rs10258925 | 0.0153 | 0.0034 | a | g | 0.2709 |
|  | rs11118120 | -0.0209 | 0.0047 | a | c | 0.8498 |
| Myristoleic acid (14:1) | rs677603 | 0.0224 | 0.004 | t | c | 0.2536 |
|  | rs11728793 | -0.021 | 0.0042 | t | c | 0.7845 |
|  | rs9888349 | -0.0192 | 0.004 | t | c | 0.7742 |
|  | rs1107722 | 0.0491 | 0.0103 | a | g | 0.0323 |
| Oleic acid (18:1) | rs11719883 | -0.0191 | 0.0034 | c | g | 0.8444 |
|  | rs6453591 | 0.0133 | 0.0025 | a | t | 0.771 |
|  | rs11133892 | 0.012 | 0.0023 | a | g | 0.4423 |
|  | rs13019537 | 0.0157 | 0.0032 | c | g | 0.841 |
| Palmitoleic acid (16:1) | rs677603 | 0.022 | 0.0042 | t | c | 0.2538 |
|  | rs11728793 | -0.0215 | 0.0044 | t | c | 0.7844 |
|  | rs1922578 | -0.0653 | 0.0142 | a | g | 0.9821 |
|  | rs13019537 | 0.0228 | 0.005 | c | g | 0.8416 |
| SNP: single-nucleotide polymorphism, EA: effect allele; OA: other allele, EAF: effect allele frequency; GX: the per-allele effect on standard deviation units; GX SE: standard error of GX. | | | | | | |
